# Impact of deceased donor acute kidney injury (AKI) on renal transplant outcomes

**DOI:** 10.1016/j.sopen.2025.11.001

**Published:** 2025-11-24

**Authors:** Mikhail Nozdrin, Maria Irene Bellini, Maria Selyanina, Maria Nozdrina, Kavyesh Vivek, Simona Mihalikova, Vassilios Papalois

**Affiliations:** a Barts NHS Foundation Trust London, United Kingdom; b Sapienza University Rome, Italy; c Queen Mary University London, United Kingdom; d Oxford University, United Kingdom; e Imperial College NHS Trust London, United Kingdom

## Abstract

**Aims:**

Donor AKI is a common reason for discarding deceased donor kidneys due to uncertainty regarding transplant outcomes. Our study investigated the effect of AKI in donor kidneys on post-transplantation outcomes.

**Methods:**

Medline, Embase, Cochrane and Web of Science were searched. Risk of bias assessment was performed. 2984 studies were identified by the search, 34 met the inclusion criteria. A total of 103,529 kidney transplants were analysed, 97,165 (94 %) with and 6364 (6 %) without donor AKI.

**Results:**

There was no significant difference between recipients of grafts from donors with terminal serum creatinine >2.0 mg/dl and < 2.0 mg/dl in 1 year serum creatinine (MD: -0.01, CI: −0.09-0.07, *P* = 0.84), 1 year patient survival (RR: 0.99, CI: 0.96–1.02, *P* = 0.52), as well as in 1 year (RR: 1.01, CI: 0.98–1.03, *P* = 0.61) and 5 year (RR: 0.99, CI: 0.94–1.04, *P* = 0.63) graft survival. DGF was the only parameter significantly worse in recipients of grafts from donors with terminal serum creatinine >2.0 than to non-AKI recipients (RR: 1.89, CI: 1.64–2.17, *P* < 0.01). In studies that compared the severity of AKI stage using the AKIN criteria, there was no significant difference in 1 year post-transplantation serum creatinine even between recipients of grafts from the most severe AKI stage (AKIN3) and the non-AKI group (AKIN0) (MD: -0.01, CI:-0.17–0.16, *P* = 0.92).

**Conclusions:**

Donor AKI is associated with a higher incidence of DGF but has no effect on post-transplant patient and graft survival and, based on this analysis, should not be a sole reason for discarding kidneys.

## Graphical abstract

**Figure:**
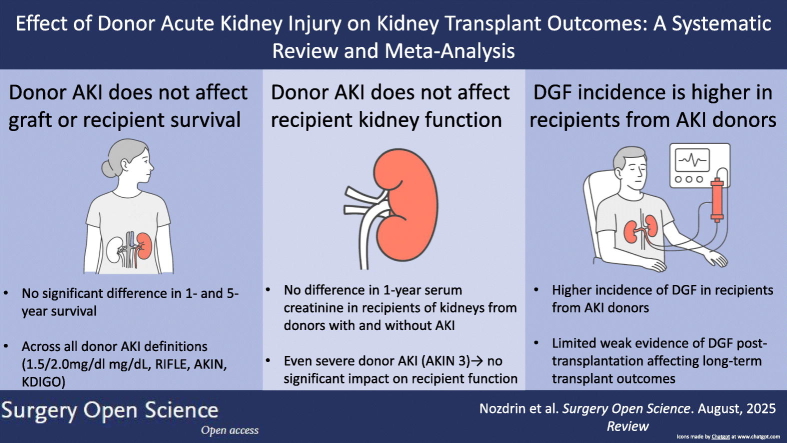

## Abbreviations

AKI: acute kidney injury
CKD: chronic kidney disease
MD: mean difference
RR: risk ratio
HR: hazard ratio
DGF: delayed graft function
PNF: primary non-function
ESRD: end stage renal disease
NHSBT: national health service blood and transplant
OPTN: organ procurement and transplantation network
KDPI: kidney donor profile index
DD: deceased donor
DCD: donor after cardiac death
DBD: donor after brain death
eGFR: estimated glomerular filtration rate
RIFLE: risk injury failure loss end-stage kidney disease
AKIN: acute kidney injury network
KDIGO: kidney disease improving global outcomes
UTI: urinary tract infection

## Introduction

Kidney transplantation in eligible patients provides superior quality of life and patient survival when compared to other forms of renal replacement therapy [1,2,8]. Growth in the number of patients with End Stage Renal Disease (ESRD) awaiting a renal transplant has outpaced the availability of organs available for transplantation [3]. Around 5000 patients in the UK are awaiting a renal transplant according to NHS Blood and Transplant (NHSBT) data [4], 40000 patients in Europe according to EU parliament sources [5] and 92,000 patients in USA according to Organ Procurement and Transplantation Network (OPTN) data [6]. The waiting time ranges from 2 to 5 years [5,9] and patients on the transplant waiting list have an annual death rate of 5 % [9].

Acute kidney injury (AKI) is common in deceased donors [10]. It can be the result of chronic kidney disease (CKD) in the donor (which in turn can be known or unknown in the donor's past medical history) and/or of acute pathology in the build-up to donation such as sepsis or hypotension [10]. AKI in younger and physically fit donors can be precipitated by vasopressin fluctuations during brain death, which in turn can precipitate haemodynamic changes [68] associated with CKD [[69], [70], [71]].

Terminal serum creatinine is a key factor in the Kidney Donor Profile Index (KDPI), used to predict graft quality and longevity, with higher KDPI scores indicating worse outcomes. Elevated terminal donor serum creatinine increases KDPI, reducing kidney utilisation chances [11]. A recent study reports US transplant surgeons are more likely to discard deceased donors with AKI compared to those without (30 % vs. 18 %) [11]. Utilizing kidneys from deceased donors (DD) with AKI could further help bridge the supply-demand gap in renal transplantation.

Transplanting kidneys from donors with AKI remains controversial. Growing evidence supports non-inferior long-term graft survival [6,7,27,28], despite the expected rise in the incidence of delayed graft function (DGF).

The aim of our meta-analysis was to investigate the effect of severity of AKI in donors, using all the major AKI classification criteria, on the post-transplantation outcomes in the recipient in both short, medium, and long-term.

## Methods

Before the study began, the trial was registered with PROSPERO CRD: 42022374136.

MEDLINE and EMBASE databases were searched through Ovid using an algorithm *(see Appendix 2)*. The search strategy was adapted for CENTRAL and Web of Science engines. Web of Science engine was used to search through the following databases: Web of Science Core Collection, BIOSIS Citation Index, CABI, KCI- Korean Journal Database, SciELO.

Our search strategy identified 2314 papers. The search results were pooled into Ovid, where duplicates and articles not in English were removed. Studies were then screened by 2 independent researchers (MN and MIB), initially by titles then abstracts and then whole papers. When a disagreement on whether a study should be included in the review occurred a 3rd reviewer (VP) was asked to settle the query.

Inclusion criteria for our study were: all papers published in English, comparing post-transplantation outcomes in adult human recipients of kidney grafts from donors with AKI present at the time of donation versus donors without AKI at the time of donation.

Papers that investigated transplantation in children and animal models were excluded. Only original research was included. Although no restriction was placed on the type of study included, the only studies that met the inclusion criteria were prospective and retrospective cohort studies.

Definitions of AKI that were accepted for our review included:

• Serum creatinine in donors greater than 1.5 mg/dl pre-donation/ retrieval, a historical threshold, before standardised definitions of AKI were agreed. Although not part of the modern consensus definitions of AKI it persists as a legacy clinical marker and a practical screening tool where true baseline donor creatinine is not known [[12], [13], [14], [15], [16]].
• Serum creatinine in donors greater than 2.0 mg/dl pre-donation/ retrieval versus, a higher threshold used in literature to identify AKI donor kidneys [13,14,[16], [17], [18], [19], [20], [21], [22], [23], [24], [25], [26]]
• RIFLE criteria (2004), developed by the Acute Dialysis Quality Initiative (ADQI) workgroup to address the lack of a uniform AKI definition. RIFLE criteria combine changes in serum creatinine or eGFR with urine output thresholds to stage AKI severity. The first three categories—Risk, Injury, and Failure—reflect progressively worsening acute renal dysfunction, while the latter two—Loss and End-stage—describe outcomes of prolonged kidney failure. RIFLE was the first widely adopted consensus definition for AKI and laid the foundation for subsequent modifications such as the AKIN and KDIGO criteria.
• AKIN criteria (2007), were developed as a refinement of the earlier RIFLE criteria. Like RIFLE, they use serum creatinine and urine output to stage severity, but they shortened the timeframe to 48 h and removed the GFR component to improve applicability. Severity is staged from Stage 1 (mild) to Stage 3 (severe, requiring renal replacement therapy).
• KDIGO criteria (2012), were developed by harmonising and unifying the earlier RIFLE and AKIN criteria to resolve discrepancies and provide a single consensus definition. Severity is staged from Stage 1 (mild) through Stage 3 (severe, potentially requiring renal replacement therapy), based on creatinine rise, GFR decrease, or duration/severity of oliguria.

A separate sub-analysis was carried out for each of the criteria above.

Outcomes investigated in our meta-analysis and systematic review included: incidence of DGF, recipient and graft survival at 1, 5 and 10-years post-transplantation, serum creatinine at 1, 5 and 10-years post-transplantation.

Risk of bias assessment was performed using NHLBI for RCT and cohort studies *(see Appendix 3).*

Data analysis and graphs were made in Revman 5.4. The I2 test was used to assess the heterogeneity of the data. All the analysis was performed using a randomised model to account for the effects of heterogeneity. Risk ratios were used to compare data that was discrete (survival, DGF incidence). Mean difference was used to compare the data that was continuous (serum creatinine post transplantation). 95 % CI intervals were used.

## Results

**Fig. 1:**
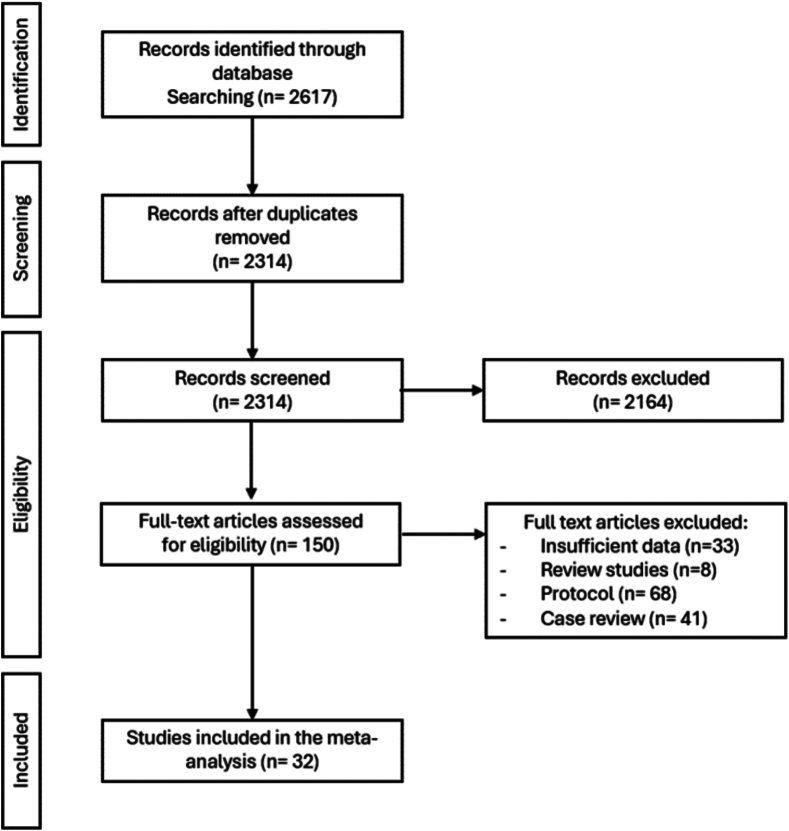
PRISMA diagram.

Our search strategy originally identified 2314 papers after removing duplicate studies. Papers were pooled into Endnote. 150 papers passed the screening by title and abstract and were used in full text assessment. 32 papers were included in the study following the screening. The total number of transplants analysed in our meta-analysis was 103,529. Flow diagram representing the screening process is demonstrated in Fig. 1.

Due to the limited number of studies examining 10-year post-transplantation graft survival, recipient survival and serum creatinine, conducting a meta-analysis on these variables was not feasible.

### Effect of terminal creatinine greater than 2.0 mg/dl in kidney donors on post-transplantation outcomes in recipients

The first AKI definition used in our study compared post-transplantation outcomes in recipients of grafts from donors with pre-donation serum creatinine >2.0 mg/dl versus <2.0 mg/dl. Thirteen papers investigated this AKI definition: [13,14,[16], [17], [18], [19], [20], [21], [22], [23], [24], [25], [26]]. Some papers reported only 1–2 outcomes and were included in respective analyses.

Eleven studies met the eligibility criteria and were included in this analysis. Recipients of grafts from donors with terminal serum creatinine lower than 2.0 mg/dl had a significantly lower incidence of DGF compared to recipients of grafts from donors with serum creatinine higher than 2.0 mg/dl. Risk ratio 1.89 (95 %CI: 1.64–2.17) p < 0.00001 [14,[16], [17], [18], [19], [20], [21], [22], [23], [24], [25], [26]]*.*

Three papers met the eligibility criteria and were included in this analysis. No significant difference between 1-year patient survival was seen between recipients of grafts from donors with serum creatinine greater than 2.0 mg/dl and serum creatinine less than 2.0 mg/dl. Risk ratio 0.99 (95 %CI: 0.96–1.02) p = 0.52 [16,18,20].

Eight papers met the inclusion criteria and were included in thiw analysis. No significant difference in 1 year graft survival was seen between recipients of grafts from donors with creatinine of greater than 2.0 mg/dl and serum creatinine less than 2.0 mg/dl. Risk ratio 1.01 (95 %CI: 0.98–1.03) p = 0.61 [13,14,[16], [17], [18], [19], [20],^23^].

Four papers met the eligibility criteria. Both recipients of grafts from donors with terminal creatinine greater than 2 mg/dl and less than 2 mg/dl had similar 1-year post-transplantation serum creatinine. Mean difference − 0.01 mg/dl (95 % CI: −0.09- 0.07) p = 0.84 [16,20,21,28]*.*

Four papers met the eligibility criteria and were included in the analysis. No significant difference was seen in 5-year graft survival between recipients of grafts from donors with serum creatinine greater than 2.0 mg/dl vs less than 2.0 mg/dl. Risk ratio: 0.99 (95 %CI: 0.94–1.04) *p* = 0.63 [13,[18], [19], [20]].

### Summary of comparison of outcomes in recipients of renal grafts from donors with terminal serum creatinine greater vs less than 2 mg/dl

In comparing post-transplantation outcomes for recipients of renal grafts from donors with terminal creatinine levels >2 mg/dl versus <2 mg/dl, the analysis of 13 papers revealed no significant differences in one-year patient and graft survival rates or five-year graft survival rates. Recipients from donors with higher terminal creatinine levels exhibited comparable one-year post-surgery serum creatinine levels but had a significantly higher incidence of DGF. These findings suggest that medium- and long-term survival and kidney function outcomes are similar between recipients of grafts from donors with terminal creatinine >2.0 mg/dl and those with <2.0 mg/dl (see Table 1).

**Table 1:** Summary of comparison of outcomes in recipients of renal grafts from donors with terminal serum creatinine greater vs less than 2 mg/dl.

Definition of AKI	Terminal serum creatinine ≥2.0 mg/dl				
# of Papers	13				
Comparison	Outcome	Findings	Statistical test (95 % CI)	*p*-value	# of papers
	Incidence of DGF	Higher incidence in grafts from AKI donors	RR: 1.89 (1.64–2.17)	<0.00001	11
Recipients of grafts from donors with terminal serum creatinine of >2.0 mg/dl vs <2.0 mg/dl	1 year recipient survival	No significant difference	RR: 0.99 (0.96–1.02)	0.52	3
	1 year graft survival	No significant difference	RR: 1.01 (0.98–1.03)	0.61	8
	1 year serum creatinine	Similar results	Mean difference − 0.01 mg/dl (−0.09–0.07)	0.84	4
	5 year graft survival	No significant difference	RR: 0.99 (0.94–1.04)	0.63	4

### Effect of terminal creatinine greater than 1.5 mg/dl in kidney donors on post-transplantation outcomes in recipients

Five studies compared recipients of grafts from donors with terminal serum creatinine greater than 1.5 mg/dl versus less than 1.5 mg/dl met the eligibility criteria and were included in the analysis. These included: [[12], [13], [14], [15], [16]]*.* Some of the papers only reported 1–2 outcomes out of all the outcomes investigated in our study and were included in respective analyses.

Three studies met the eligibility criteria and were included in the analysis. No significant difference was seen between 1 year patient survival in recipients of grafts from serum creatinine greater than 1.5 mg/dl versus less than less than 1.5 mg/dl. Risk ratio was 1.01 (95 % CI: 0.96–1.07) p = 0.63 [12,15,16].

Five studies met the eligibility criteria and were included in the analysis. No significant difference was seen between the recipients of grafts from donors with serum creatinine. Risk ratio was 1.00 (95 %CI: 0.99–1.00) *p* = 0.19 [[12], [13], [14], [15], [16]]*.*

### Summary of comparison of outcomes in recipients of renal grafts from donors with terminal serum creatinine greater vs less than 1.5 mg/dl

In evaluating post-transplantation outcomes for recipients of renal grafts from donors with terminal serum creatinine levels >1.5 mg/dl versus <1.5 mg/dl, data from five studies were analysed. No significant differences were found in one-year patient survival (RR 1.01; 95 % CI: 0.96–1.07; *p* = 0.63) or one-year graft survival (RR 1.00; 95 % CI: 0.99–1.00; *p* = 0.19) with terminal creatinine of <1.5 mg/dl vs >1.5 mg/dl. These findings indicate that medium-term survival outcomes are similar for recipients of grafts from donors with terminal creatinine levels >1.5 mg/dl compared to those with <1.5 mg/dl (see Table 2).

**Table 2:** Summary of comparison of outcomes in recipients of renal grafts from donors with terminal serum creatinine greater vs less than 1.5 mg/dl.

Definition of AKI	Terminal serum creatinine ≥ 1.5 mg/dl				
# of Papers	5				
Comparison	Outcome	Significant findings	Statistical test (95 % CI)	p-value	# of papers
Recipients of grafts from donors with terminal serum creatinine of >1.5 mg/dl vs <1.5 mg/dl	1 year recipient survival	No significant difference	RR: 1.01 (0.96–1.07)	0.63	3
	1 year graft survival	No significant difference	RR: 1.00 (0.99–1.00)	0.19	5

### Effect of terminal donor AKI using the RIFLE criteria on post-transplantation outcomes in recipients

A total of 4 studies were eligible and met the inclusion criteria. These included: [[34], [35], [36], [37]]. Some of the papers only reported 1–2 outcomes out of all the outcomes investigated in our study and were included in respective analyses.

Three studies met the eligibility criteria and were included in the analysis [[34], [35], [36]]*.* Overall, there was no significant difference in the incidence of DGF in transplant recipients of renal grafts from donors with and without AKI according to the RIFLE criteria (Risk ratio 1.20 95 % CI: 0.41–3.50 *P* = 0.73).

A total of 3 studies met the eligibility criteria and were included in the analysis. These included [34,36,37]. Overall, there was no significant difference in serum creatinine 1 year post transplantation in recipients of renal grafts from donors with and without AKI according to the RIFLE criteria (Mean Difference 0.07 95 % CI: −0.14- 0.29 *P* = 0.5).

### Summary of comparison of outcomes in recipients of renal grafts from donors with an AKI according to the RIFLE criteria

In evaluating renal transplant outcomes based on the RIFLE criteria for AKI, four studies were considered. A pooled analysis of three studies showed no significant difference in DGF incidence between recipients of grafts from donors with and without AKI (RR 1.20; 95 % CI: 0.41–3.50; *p* = 0.073). Additionally, a separate analysis of one-year post-transplant serum creatinine levels from three studies found no significant difference (mean difference 0.07; 95 % CI: −0.14-0.29; *p* = 0.5). These results suggest that donor AKI, according to RIFLE criteria, does not significantly impact the risk of DGF or one-year serum creatinine levels in renal transplant recipients (see Table 3).

**Table 3:** Summary of comparison of outcomes in recipients of renal grafts from donors with AKI classified by RIFLE criteria.

Definition of AKI	RIFLE criteria				
# of Papers	4				
Comparison	Outcome	Significant findings	Statistical test (95 % CI)	p-value	# of papers
Recipients of grafts from donors with pre-donation AKI according to RIFLE criteria vs non-AKI	Incidence of DGF	No significant difference	RR 1.20 (0.41–3.50)	0.73	3
	1 year serum creatinine	No significant difference	Mean difference 0.07 (−0.14- 0.29)	0.5	3

### Effect of terminal donor AKI using the KDIGO criteria on post-transplantation outcomes in recipients

A total of 5 studies were eligible and met the inclusion criteria [27,[38], [39], [40], [41]]. *Park, W·C* et al. [41] study included in this analysis, further stratified donors by being standard criteria and extended criteria donors. This data was presented as 2 separate sub-groups and in our analysis we included both cohorts of the study by *Park, W·C* [41] separately as they were presented in the original study. Some of the papers only reported 1–2 outcomes out of all the outcomes investigated in our study and were included in respective analyses.

Four studies met the eligibility criteria and were included in this analysis: [27,[38], [39], [40]]. A significantly lower incidence of DGF was seen in recipients of grafts from non-AKI donors (Risk ratio 2.87 95 % CI 1.27–6.49 *P* = 0.01).

Three studies met the eligibility criteria and were included in the analysis. These included: [27,40,41]. There was no significant difference in patient survival 1 year following transplantation in recipients of grafts from donors with and without AKI according to the KDIGO criteria (Risk ratio 0.99 95 %CI 0.97–1.02 *P* = 0.51).

Three studies met the eligibility criteria. These included [27,40,41]. No significant difference was seen in one year graft survival between recipients of grafts from donors with and without AKI using KDIGO criteria (Risk ratio 1.01 95 % CI: 0.98–1.04 *P* = 0.67).

Four studies met the eligibility criteria. These included: [27,[39], [40], [41]]. No significant difference was seen in five-year graft survival between recipients of grafts from donors with and without AKI using KDIGO criteria (Risk ratio 1.00 95 % CI: 0.94–1.06 *P* = 0.88).

Three studies met the eligibility criteria. These included: [27,40,41]. No significant difference was seen in five-year recipient survival between recipients of grafts from donors with and without AKI using KDIGO criteria (Risk ratio 0.97 95 % CI: 0.9–1.04 *P* = 0.37).

### Summary of comparison of outcomes in recipients of renal grafts from donors with an AKI according to the KDIGO criteria

In the comparison of outcomes for recipients of renal grafts from donors with and without AKI according to the KDIGO criteria, five eligible studies were included. No significant difference was seen in the medium- long term in both graft and recipient survival in recipients of grafts from donors with and without terminal AKI. Similarly to other sub-analyses of AKI using criteria other than KDIGO, incidence of DGF was significantly higher in recipients of grafts from donors with AKI *(see* Table 4*).*

**Table 4:** Summary of comparison of outcomes in recipients of renal grafts from donors with AKI classified by KDIGO criteria.

Definition of AKI	KDGIO criteria				
# of Papers	5				
Comparison	Outcome	Significant findings	Statistical test (95 % CI)	p-value	# of papers
	Incidence of DGF	Higher incidence in grafts from AKI donors	RR: 2.87 (1.27–6.49)	0.01	4
Donors with pre-donation AKI according to KDIGO criteria vs non-AKI	1 year recipient survival	No significant difference	RR: 0.99 (0.97–1.02)	0.37	3
	1 year graft survival	No significant difference	RR: 1.01 (0.98–1.04)	0.67	3
	5 year graft survival	No significant difference	RR: 1.00 (0.94–1.06)	0.88	4
	5 year recipient survival	No significant difference	RR: 0.97 (0.90–1.04)	0.37	3

### Effect of terminal donor AKI using the AKIN criteria on post-transplantation outcomes in recipients

Seven studies met the eligibility criteria and were included in the meta-analysis. These included: [[42], [43], [44], [45], [46], [47], [48]]*.* Some of the papers only reported 1–2 outcomes out of all the outcomes investigated in our study and were included in respective analyses.

Two studies met the eligibility criteria and were included in the analysis [42,47]:. No significant difference was seen in the recipient survival of grafts from donors with AKI per AKIN criteria vs no AKI (Risk ratio: 0.97 95 %CI: 0.91–1.04 *P* = 0.45).

Two studies met the eligibility criteria and were included in the analysis: [42,47]. Recipients of grafts without AKI per AKIN had a marginally better 1-year graft survival compared to recipient of grafts with AKI (Risk ratio 0.95 0.95 %CI: 0.91–0.99 *P* = 0.01).

Three studies met the eligibility criteria: [42,45,47]. No significant difference was seen in five-year graft survival between recipients of grafts from donors with AKI (AKIN criteria) and without AKI (Risk ratio: 0.99 95 %CI: 0.9–1.10 *P* = 0.87).

### Effect of donor AKI stage per AKIN criteria on post-transplantation serum creatinine in kidney transplant recipients 1-year post-transplantation

Three studies compared the effect of severity of donor AKI (AKIN Stage 1,2,3) vs absence of donor AKI (AKIN 0) on 1-year post-transplantation serum creatinine [42,43,48].

No significant difference was seen in recipients of kidney grafts from donors with AKIN stage 1 vs no AKIN (Risk ratio Mean difference 0.01 mg/dl 95 %CI: −0.13- 0.15 *P* = 0.88).

No significant difference was seen in recipients of kidney grafts from donors with AKIN stage 2 vs no AKIN (Risk ratio Mean difference − 0.10 mg/dl 95 %CI: −0.25- 0.06 *P* = 0.21).

No significant difference was seen in recipients of kidney grafts from donors with AKIN stage 3 vs no AKIN (Risk ratio Mean difference − 0.01 mg/dl 95 %CI: −0.17- 0.16 *P* = 0.92).

Overall, no significant difference was seen in 1 year serum creatinine in recipients of renal grafts from donors with and without AKI per AKIN criteria (Mean difference − 0.03 mg/dl 95 %CI: −0.13- 0.07).

### Effect of AKIN stage on serum creatinine 1-year post-transplantation

There was no significant difference in serum creatinine one year following transplantation in each of the AKIN1+ stages when compared to recipients of grafts from donors with no AKI per AKIN criteria, even in the most severe AKI group (AKIN 3). Thus, through an indirect comparison, it is possible to assume that as the AKI worsens in the DD kidney donor, serum creatinine in transplant recipients one year post transplantation is not affected.

### Effect of AKIN stage on incidence of delayed graft function in kidney transplant recipients

Five studies met the eligibility criteria and were included in the analysis: [[42], [43], [44], [45],^47^].

Recipients of grafts from donors with an AKI stage 1 had a marginally higher incidence of DGF compared to recipients of grafts from non-AKI donors (Risk ratio: 1.13 95 %CI: 1.00–1.27 *P* = 0.05).

Recipients of grafts from donors with an AKI stage 2 had a higher incidence of DGF compared to recipients of grafts from non-AKI donors (Risk ratio: 1.43 95 %CI: 1.04–1.95 *P* = 0.03).

Recipients of grafts from donors with an AKI stage 3 had a higher incidence of DGF compared to recipients of grafts from non-AKI donors (Risk ratio: 2.20 95 %CI: 1.95–2.48 *P* < 0.001).

Overall recipients from donors with an AKI any stage AKIN1+ had a significantly higher incidence of DGF compared to recipients of grafts from donors with no AKI (Risk ratio: 1.67 95 %CI: 1.38–2.01 *P* < 0.01).

### Summary of comparison of outcomes in recipients of renal grafts from donors with an AKI according to the AKIN criteria

In comparing outcomes for recipients of renal grafts from donors with and without AKI according to the AKIN criteria, the analysis showed no significant difference in one-year recipient survival. Recipients of grafts without AKI had marginally better one-year graft survival compared to those with AKI (RR 0.95; 95 % CI: 0.91–0.99; *p* = 0.01), but long-term 5-year graft survival showed no significant difference (RR 0.99; 95 % CI: 0.90–1.10; *p* = 0.87) (see Table 5).

**Table 5:** Summary of comparison of outcomes in recipients of renal grafts from donors with AKI classified by AKIN criteria.

Definition of AKI	AKIN criteria				
# of Papers	7				
Comparison	Outcome	Significant findings	Statistical test (95 % CI)	p-value	# of papers
	Incidence of DGF	Higher incidence in recipients of AKI donors	RR: 1.67 (1.38–2.01)	<0.00001	5
Donors with pre-donation AKI according to AKIN criteria vs non-AKI	1-year recipient survival	No significant difference	RR: 0.97 (0.91–1.04)	0.45	2
	1-year graft survival	Higher survival in non-AKI kidney recipients	RR: 0.95 (0.91–0.99)	0.01	2
	1-year serum creatinine	No significant difference	Mean difference − 0.03 mg/dl (−0.13- 0.07)	0.58	3
	5-year graft survival	No significant difference	RR: 0.99 (0.9–1.10)	0.87	3

One-year post-transplant serum creatinine levels were not significantly different in recipients of grafts even from donors with Stage 3 AKI compared to non-AKI donors (see Table 6). However, AKIN1+ donors had a significantly higher incidence of DGF, with increasing risk ratios for higher AKI stages (AKIN1 vs. AKIN0 RR: 1.13; AKIN2 vs. AKIN0 RR: 1.43; AKIN3 vs. AKIN0 RR: 2.20), suggesting that DGF incidence escalates with worsening AKI severity (see Table 7).

**Table 6:** Summary of effects of AKI stage (AKIN criteria) on DGF.

Definition of AKI	AKIN criteria				
# of Papers	4				
	Comparison	Significant findings	Statistical test (95 % CI)	p-value	# of papers
Incidence of DGF	AKI stage 0 vs AKI stage 1	No significant difference	RR: 1.13 (0.91–1.04)	0.05	4
	AKI stage 0 vs AKI stage 2	Higher DGF in recipients of AKI kidneys	RR: 1.43 (1.04–1.95)	0.03	4
	AKI stage 0 vs AKI stage 3	Higher DGF in recipients of AKI kidneys	RR: 2.20 (1.95–2.48)	<0.00001	4

**Table 7:** Summary of effects of AKI stage (AKIN criteria) on 1-year serum creatinine.

Definition of AKI	AKIN criteria				
# of Papers	3				
	Comparison	Significant findings	Statistical test (95 % CI)	p-value	# of papers
Serum creatinine at 1 year	AKI stage 0 vs AKI stage 1	No significant difference	Mean difference 0.01 mg/dl (−0.13- 0.15)	0.88	3
	AKI stage 0 vs AKI stage 2	No significant difference	Mean difference − 0.1 mg/dl (−0.25- 0.06)	0.21	3
	AKI stage 0 vs AKI stage 3	No significant difference	Mean difference − 0.01 mg/dl (−0.17- 0.16)	0.92	3

## Discussion

A key strength of this study lies in its comprehensive analysis of data across multiple established definitions of acute kidney injury (AKI), including RIFLE, AKIN, KDIGO, and serum creatinine thresholds. While these definitions aim to identify and classify the same clinical condition, they vary in the parameters considered and the thresholds applied, which could influence the identification and grading of AKI. By utilizing all these definitions, we sought to determine whether a universal trend in post-transplant outcomes could be observed regardless of how AKI was defined. This approach addresses potential concerns regarding the selection of a single definition, which could introduce bias or limit the generalizability of the findings. Importantly, our analysis demonstrated that appropriately selected donor kidneys with AKI perform well post-transplant, irrespective of the specific criteria used to define AKI. These findings underscore the robustness of our results and contribute to a broader understanding of the implications of donor AKI in kidney transplantation.

While this study primarily focuses on the impact of AKI on donor kidney outcomes, it is important to acknowledge that the selection of donor kidneys for transplantation involves numerous factors beyond serum creatinine levels. The individual studies included in this meta-analysis employed strict selection criteria tailored to the specific centres and countries where they were conducted, which likely accounted for additional donor characteristics. By synthesising data from these diverse studies, the meta-analysis methodology inherently mitigates the biases introduced by the variability in individual study inclusion and exclusion criteria. This approach reduces the impact of heterogeneity and ensures a more robust and generalizable assessment of the performance of AKI-affected kidneys compared to those without AKI. Consequently, the findings provide valuable insights into the potential of appropriately selected donor kidneys with AKI to achieve comparable post-transplant outcomes, despite the inherent variability in donor selection practices across studies.

### Graft survival and patient survival

Across all our analyses, no statistical difference was found in 1-year and 5-year patient and graft survival between recipients of grafts from donors with and without AKI. Nita et al. [32] showed similar findings (OR 0.95, *p* = 0.54), despite using a single analysis for various time endpoints.

The majority of registry data supports the findings of our meta-analysis [30,31]. *Liu* et al [49], utilised data from OPTN and analysed outcomes in 25,323 recipients (between 2010 and 2013) found no association in donor AKI status (KDIGO criteria) with death-censored graft failure (hazard ratio, 1.01; 95 % CI, 0.95–1.08) or all-cause graft failure (hazard ratio, 0.97; 95 % CI, 0.93–1.02).

A Korean national database study using definition of an AKI as terminal serum creatinine >2.0 mg/dl, analysed outcomes in 1466 kidney transplants, and found no significant difference in overall graft failure (HR 0.973; 95 % CI 0.584–1.621), death-censored graft failure (HR 1.004; 95 % CI 0.491–2.054), and mortality (HR 0.808; 95 % CI 0.426–1.532) based on donor AKI status (50).

UK registry data by Boffa et al. [29] using the AKIN definition of AKI in 11,649 patients (2003−2013) showed no significant difference in graft failure/death at 90 days, but a higher rate at 1 year (9.1 % AKI vs. 10.8 %, *p* = 0.02, OR 1.2, 95 % CI 1.03–1.41). Boffa et al. [29] questioned the clinical significance, noting the annual death rate risk on the transplant waiting list was significantly higher than the risk from transplanted AKI donor kidneys.

### Graft function: short-term, medium-term, and long-term

#### Delayed graft function

DGF is a manifestation of AKI post-transplantation, occurring in about 20 % of DD transplants [51]. DGF in renal transplantation is often defined as serum creatinine >3.0 mg/dl or the need for dialysis within a week post-surgery, though definitions in literature vary and can be extended to 28 days, causing analysis bias.

**Fig. 2:**
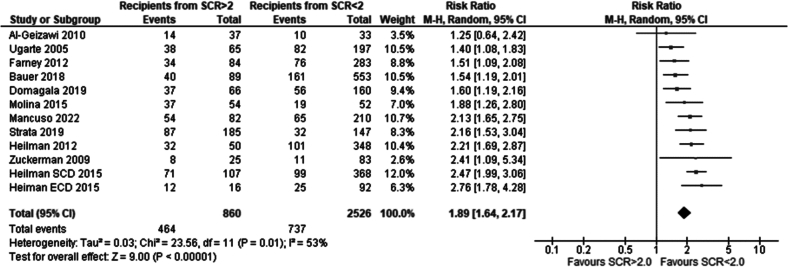
Incidence of delayed graft function in recipients of renal grafts from donors with terminal creatinine greater than 2 mg/dl vs less than 2 mg/dl.

**Fig. 3:**
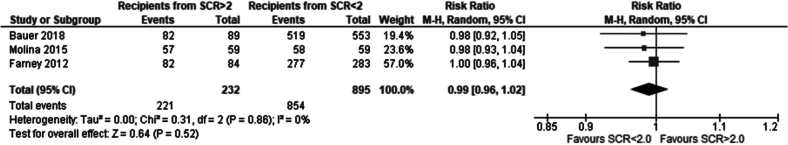
One year patient survival for recipients of renal grafts from donors with terminal creatinine greater than 2 mg/dl vs less than 2 mg/dl.

**Fig. 4:**
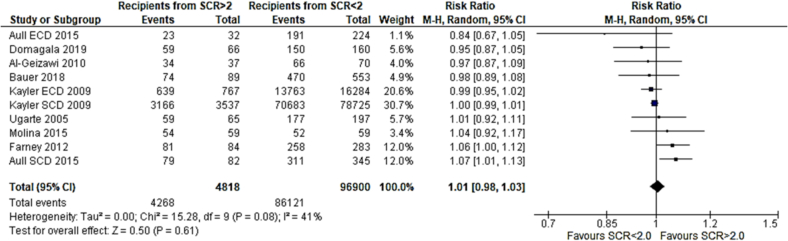
One year graft survival for recipients of renal grafts from donors with terminal creatinine greater than 2 mg/dl vs less than 2 mg/dl.

Our study has shown an overall significantly higher incidence of DGF in recipients of grafts from donors with AKI than without AKI. In the analysis comparing graft recipients with terminal donor creatinine >2.0 mg/dl to those with levels <2.0 mg/dl (Fig. 2), DGF likelihood was 1.89 times higher, with 29 % incidence in non-AKI and 54 % in AKI groups. Recipients with RIFLE-defined AKI showed no significant DGF difference (Fig. 9), with a 26 % incidence in RIFLE AKI and 43 % in non-AKI groups. KDIGO-defined AKI increased DGF risk by 2.87 times (Fig. 11), with 25 % incidence in AKI versus 14 % in non-AKI groups. AKIN-defined AKI increased DGF risk by 1.67 times (Fig. 26), with 47 % incidence in AKI versus 26 % in non-AKI groups. (See Fig. 3, Fig. 4.)

Our study demonstrates donor AKI severity significantly affects DGF development, through the AKIN sub-analysis. AKIN1 recipients showed no significant DGF difference (Fig. 23) compared to AKIN0, AKIN2 recipients showed a marginally significant increase (RR 1.43; 95 % CI 1.04–1.95; *p* = 0.04) (Fig. 24) whilst AKIN3 recipients had a 2.2-fold increase (RR 95 % CI: 1.95–2.48; *p* < 0.001) in DGF incidence (Fig. 25). An inference can be made that DGF expectedly increases with AKI severity, as pre-existing AKI is exacerbated by inevitable ischaemic/reperfusion injury during transplantation.

The long-term effects of DGF are debated. A meta-analysis by Li et al. [52] found higher acute rejection (OR 1.84; 95 % CI, 1.30–2.61; *P* < 0.01), 1-year recipient mortality (OR 2.32; 95 % CI, 1.53–3.50; P < 0.01), and graft failure (OR 3.38; 95 % CI, 1.85–6.17; P < 0.01) with DGF, while other studies [[53], [54], [55], [56], [57]] showed no significant differences. The significance of DGF on long-term graft function remains controversial [52].

The duration of DGF is an important factor to consider when examining the significance of DGF on long-term post-transplantation outcomes. The UK registry study [58] only showed a significant difference in death censored graft failure between recipients of grafts from AKI and non-AKI donors when the duration of DGF was greater than 14 days in recipients of AKI grafts. The Australian study found a direct time dependent link between duration of DGF and graft loss [59].

Locke et al. [72] study using UNOS data, compared DGF incidences in kidneys from donors after cardiac death (DCD) and brain death (DBD), showing higher DGF rates in DCD kidneys (38.7 %) compared to SCD (19.5 %) and ECD (30 %) DBD kidneys. Despite higher DGF in DCD kidneys, those from donors under 50 years had similar long-term graft survival to SCD kidneys if cold ischemic time was under 12 h, indicating DGF impact can be mitigated with optimal practices [72].

#### Serum creatinine

We found no significant difference in post-transplant serum creatinine between recipients of grafts from donors with and without AKI across all analyses: Serum Creatinine >2 mg/dl vs <2 mg/dl (Fig. 5); RIFLE AKI vs non-AKI (Fig. 10); AKIN AKI vs non-AKI (Fig. 22); and all AKIN sub-analyses (Fig. 19, Fig. 20, Fig. 21). (See Fig. 6, Fig. 7, Fig. 8.) (See Fig. 12, Fig. 13, Fig. 14, Fig. 15, Fig. 16, Fig. 17, Fig. 18.)

**Fig. 5:**
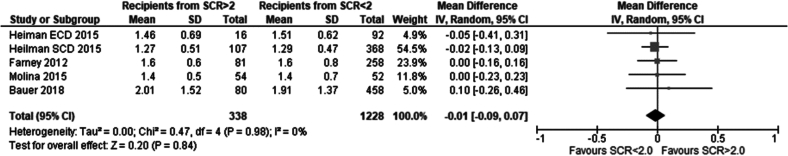
One year post-transplant serum creatinine in recipients of renal grafts from donors with terminal creatinine greater than 2 mg/dl vs less than 2 mg/dl.

**Fig. 9:**
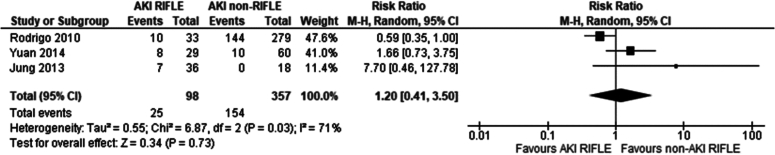
Incidence of delayed graft function post-transplantation in recipients of grafts from donors with AKI, determined by the RIFLE criteria vs recipients without AKI.

**Fig. 10:**
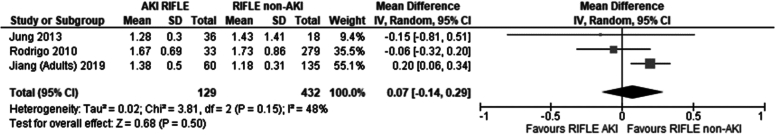
One year serum creatinine in recipients of grafts from donors with AKI, determined by the RIFLE criteria vs recipients without AKI.

**Fig. 11:**
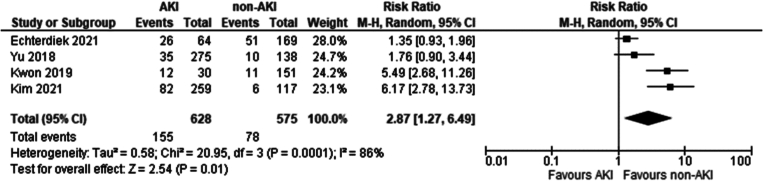
Incidence of Delayed Graft Function post-transplantation in recipients of grafts from donors with AKI, determined by the KDIGO criteria vs recipients without AKI.

**Fig. 19:**
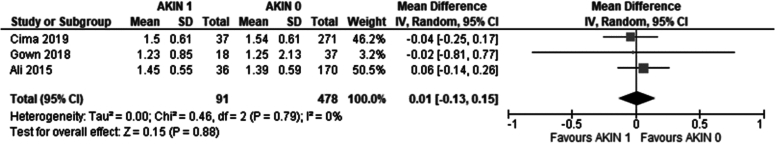
One-year serum creatinine in transplant recipients from donors with AKIN1 AKI vs no AKI per AKIN criteria.

**Fig. 20:**
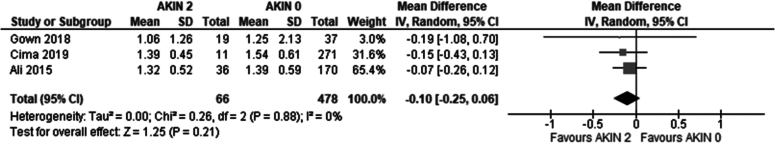
One year serum creatinine in transplant recipients from donors with AKIN2 AKI vs no AKI per AKIN criteria.

**Fig. 21:**
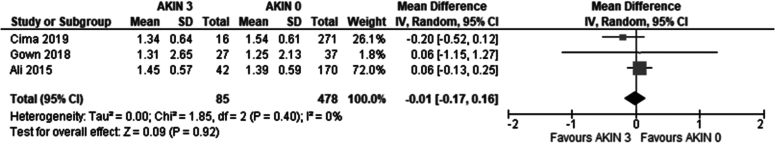
One-year serum creatinine in transplant recipients from donors with AKIN3 AKI vs no AKI per AKIN criteria.

**Fig. 22:**
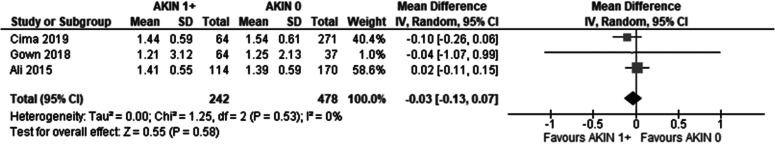
One-year serum creatinine in transplant recipients from donors with all stages of AKI per AKIN criteria vs no AKI per AKIN criteria.

**Fig. 23:**
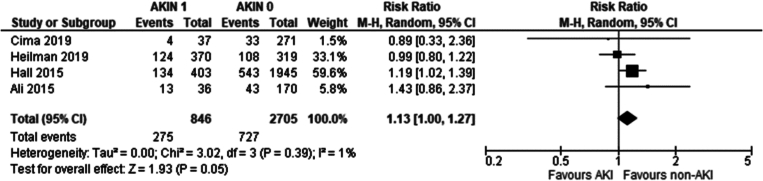
Delayed graft function in transplant recipients from donors with AKIN1 AKI vs no AKI per AKIN criteria.

**Fig. 24:**
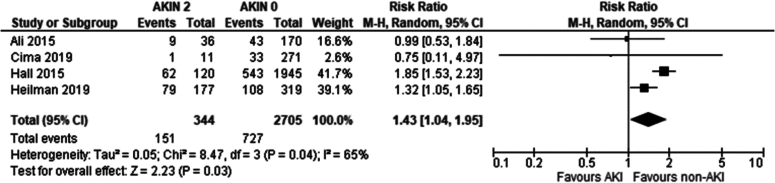
Delayed graft function in transplant recipients from donors with AKIN2 AKI vs no AKI per AKIN criteria.

**Fig. 25:**
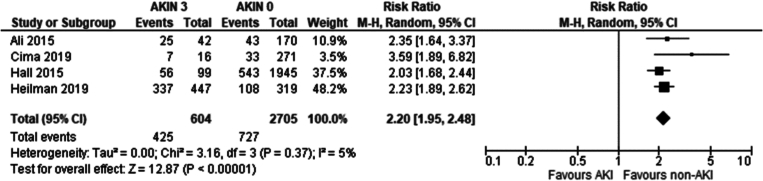
Delayed graft function in transplant recipients from donors with AKIN3 AKI vs no AKI per AKIN criteria.

**Fig. 26:**
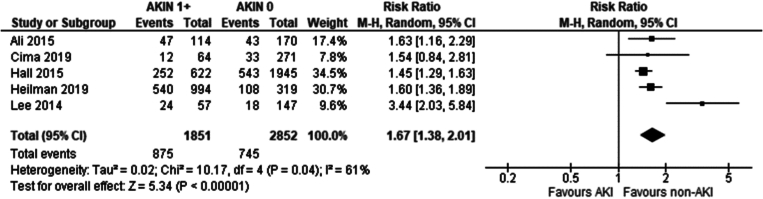
Delayed graft function in transplant recipients from donors with all stages of AKI per AKIN criteria vs no AKI per AKIN criteria.

**Fig. 6:**
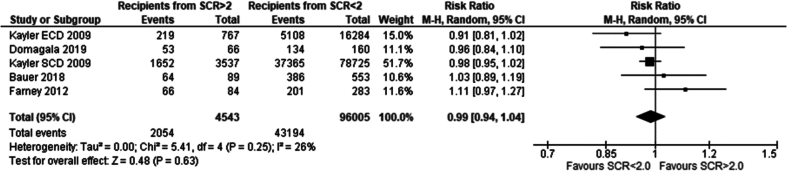
Five-year graft survival for recipients of renal grafts from donors with terminal creatinine greater than 2 mg/dl vs less than 2 mg/dl.

**Fig. 7:**
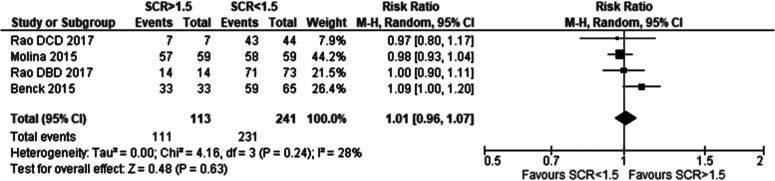
One year patient survival in recipients of renal grafts from donors with terminal creatinine greater than 1.5 mg/dl vs less than 1.5 mg/dl.

**Fig. 8:**
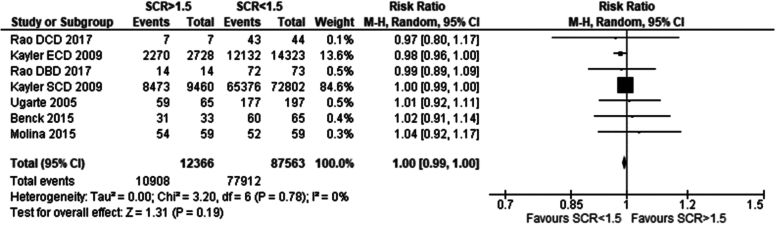
One year graft survival in recipients of renal grafts from donors with terminal creatinine greater than 1.5 mg/dl vs less than 1.5 mg/dl.

**Fig. 12:**
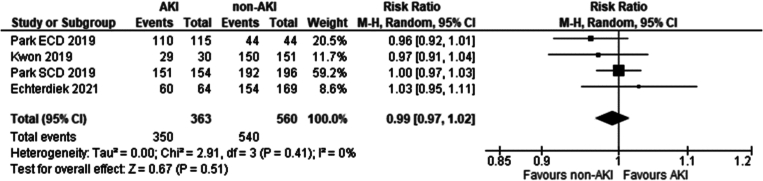
One year recipient survival in recipients of grafts from donors with AKI, determined by the KDIGO criteria vs recipients without AKI.

**Fig. 13:**
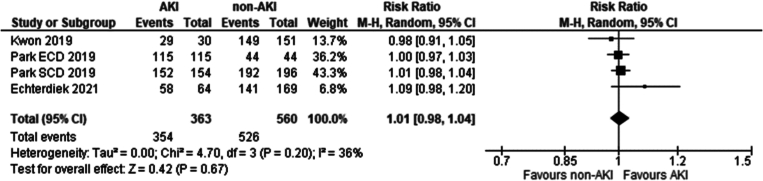
One year graft survival in recipients of grafts from donors with AKI, determined by the KDIGO criteria vs recipients without AKI.

**Fig. 14:**
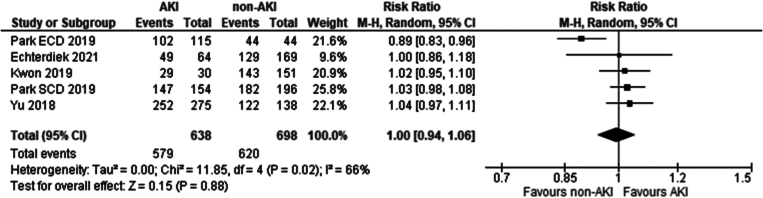
Five-year graft survival in recipients of grafts from donors with AKI, determined by the KDIGO criteria vs recipients without AKI.

**Fig. 15:**
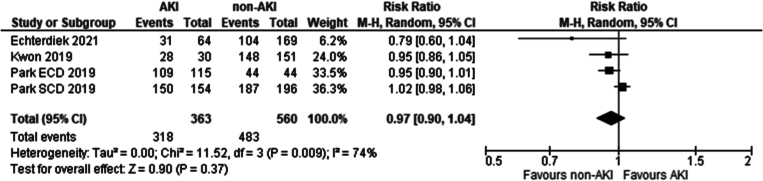
Five-year patient survival in recipients of grafts from donors with AKI, determined by the KDIGO criteria vs recipients without AKI.

**Fig. 16:**
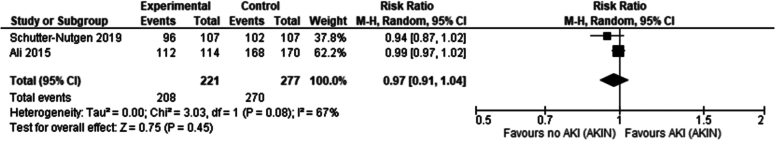
One-year recipeint survival in recipients of grafts from donors with AKI, determined by the AKIN criteria vs recipients without AKI.

**Fig. 17:**
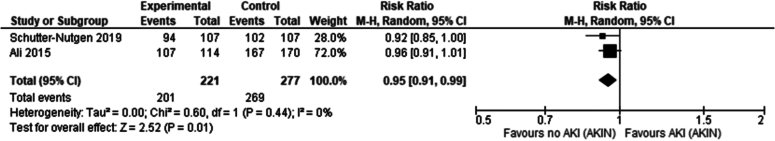
One year graft survival in recipients of grafts from donors with AKI, determined by the AKIN criteria vs recipients without AKI.

**Fig. 18:**
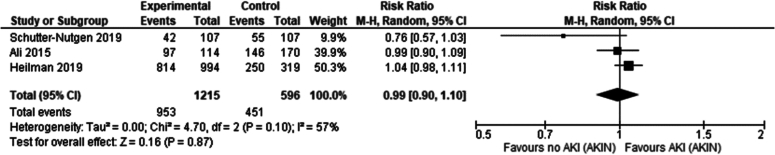
Five-year graft survival in recipients of grafts from donors with AKI, determined by the AKIN criteria vs recipients without AKI.

Serum creatinine post transplantation is an important surrogate marker in predicting long-term graft function and survival [[60], [61], [62]]. One year serum creatinine post-transplantation has been shown to be a good predictor of long-term graft function [63].

Our meta-analysis aligns with registry data. Ha, J. et al. [50] found no difference in one-year serum creatinine in recipients of grafts from AKI (donor creatinine >2.0 mg/dl) vs non-AKI donors in the Korean transplant registry. The UK registry study by *Boffa, C.* et al [29], which used the AKIN criteria to classify donor AKI, showed a statistically significant difference in one-year post-transplantation serum creatinine in recipients of grafts from AKI vs non-AKI donors.

The analysis by *Boffa, C. et al* [29] split the 1-year post transplantation serum creatinine outcome in recipients into discrete groups of: >60, 45–60, 30–45, <30, return to dialysis. There was minimal difference of 1–2 % between prevalence of recipients of grafts from non-AKI vs AKI donors in each of the yearly serum creatinine categories. In the return to dialysis outcome, the difference was around 0.5 %. No analysis was performed investigating whether there was a statistically significant difference between the prevalence of patients who had received a graft from AKI vs non-AKI donor in each of the outcome groups. Due to these factors, the statistically significant worse serum creatinine in recipients of grafts from donors with AKI likely has minimal clinical significance.

The largest US registry study by Liu, C. et al. [49] compared 6-month eGFR using KDIGO AKI definitions and found statistically significant but not clinically meaningful differences (*P* < 0.001) among AKI stages, with mean 6-month eGFRs of 61, 58, 57, and 62 ml/min/1.73 m^2^ for no AKI, stage 1, stage 2, and stage 3, respectively. Like the largest registry study, our meta-analysis shows no significant difference in the serum creatinine levels one-year post-transplantation using AKIN definition of AKI.

### The utilisation of AKI stage 3 kidneys

Most data, including large registry studies and our meta-analysis, support using kidneys from DD with terminal AKI stages 1 and 2 [29,49]. However, AKI stage 3 kidneys remain controversial.

The US registry study by Liu et al. [49] analysed 1273 recipients of AKI stage 3 kidneys using KDIGO criteria, comparing them to non-AKI DD recipients. They found no significant difference in death-censored graft failure (HR: 0.90 CI 0.77–1.06) but reported higher all-cause graft failure (HR: 0.85 CI 0.75–0.95) and worse, though not clinically significant, creatinine levels and higher DGF incidence.

The UK registry study by Boffa et al. [29] analysed 172 AKIN stage 3 AKI recipients, finding a higher incidence of primary non-function (PNF) (9 % vs. 4 %; OR 3.09 CI 1.54–6.16) and DGF, but did not comment on serum creatinine or long-term graft survival.

Lapparisuth et al. [33], using the Thailand Transplant registry, analysed 4234 kidney transplants (2001–2018) with KDIGO AKI classification. They found no significant difference in recipient survival for AKI stage 3 grafts compared to other stages (HR 0.79 CI 0.60–1.05) and no overall difference between AKI and non-AKI grafts (*p* = 0.69). However, persistent AKI in stage 3 grafts was a significant risk factor for graft loss.

All three registry studies advise caution with AKI stage 3 kidneys. The Thai study linked increased transplant failure in AKI stage 3 recipients with persistent AKI to infection risk [33]. DGF is associated with early post-transplant infections, including UTI and BK Viremia [63], and secondary infections [64].

The UK study noted a higher incidence of PNF but did not comment on DGF duration. Persistent AKI post-transplant is associated with immune activation, leading to inflammatory cytokine production and organ damage [65]. The combination of pre-existing AKI, ischemic injury, and immune injury likely contributes to the higher PNF incidence in stage 3 AKI donors.

Our meta-analysis shows long-term comparable kidney function in AKI stage 3 donors, while registry data indicates marginally worse early allograft survival. Strategies to mitigate early graft loss, such as infection monitoring and immunosuppression adjustments, are essential. The Thai registry emphasised infection as a major cause of graft loss in stage 3 AKI kidneys. Careful recipient selection and optimization, as highlighted by the US study, can improve outcomes. Machine perfusion trials for stage 3 AKI kidneys have shown reduced DGF and better long-term outcomes compared to static cold storage [66].

The slightly reduced graft survival in AKI stage 3 kidneys should be weighed against the high mortality rate on the transplant waiting list (annual death rate of 5 %) [9]. The 20 % increased graft failure risk with AKI stage 3 is comparable to the 17 % increased risk with six months of dialysis compared to pre-emptive transplantation and significantly lower than the 37 % and 55 % increased risks with one or two years of dialysis before transplantation [67].

### Limitations

Analysing the effects of AKI presence vs. absence using various criteria (RIFLE, KDIGO, AKIN) or serum creatinine thresholds (1.5 mg/dl or 2.0 mg/dl) is biased, as the AKI group includes a spectrum of severity, from minimal to severe AKI. The meta-analysis quality heavily depends on patient selection in the original studies. To counteract this bias, we performed sub-analyses by AKI severity (AKIN sub-analyses), where recipients were stratified by AKI severity. However, unfortunately there were insufficient studies using RIFLE and KDIGO criteria to conduct a systematic review or meta-analysis.

## Conclusion

In the present study, AKI effects on post-transplant outcomes were meta-analysed, comparing AKI presence using major classifications and stratifying AKI severity where possible. No statistically significant differences in medium-term (1-year) and long-term (5-years) graft and patient survival was observed. Serum creatinine levels at 1 and 5 years were also comparable, serving as a predictor of long-term graft function.

However, our findings showed a significantly higher incidence of DGF in recipients of grafts from AKI donors. The severity of donor AKI significantly led to DGF development, with varying risks across AKI stages. Despite this, the clinical significance of DGF remains debated, as patient survival, graft survival, and serum creatinine levels were similar between AKI and non-AKI recipients, suggesting the increased DGF incidence may be non-significant.

Recipients of grafts from AKI stages 1 and 2 have outcomes comparable to non-AKI graft recipients. While AKI stage 3 graft recipients showed marginally worse graft survival, we suggest to still use them, as they offer better patient survival compared to dialysis.

In conclusion, our meta-analysis confirms what most registry studies also suggest - that kidneys from donors with terminal AKI are not inferior to those from non-AKI donors and should not be discarded on this basis.

## CRediT authorship contribution statement

**Mikhail Nozdrin:** Writing – review & editing, Writing – original draft, Software, Project administration, Methodology, Investigation, Formal analysis, Data curation, Conceptualization. **Maria Irene Bellini:** Writing – review & editing, Supervision, Data curation. **Maria Selyanina:** Writing – original draft, Data curation. **Maria Nozdrina:** Writing – original draft, Formal analysis. **Kavyesh Vivek:** Writing – review & editing, Formal analysis. **Simona Mihalikova:** Writing – original draft, Data curation. **Vassilios Papalois:** Writing – review & editing, Writing – original draft, Supervision, Methodology, Investigation, Conceptualization.

## Ethics approval

As this study is a systematic review and meta-analysis of previously published data, ethical approval and patient consent were not required.

## Funding sources

This research received no external funding and was conducted without financial support from any organization.

## Declaration of competing interest

The authors declare that they have no competing interests. No author has received sponsorship, consultancy fees, or any form of financial support from commercial organizations.

## Appendix A Supplementary data

Appendix 1 Acute Kidney injury classifications. Image 1

Appendix 2 Embase & Ovid MEDLINE(R) Search strategy Image 2

Appendix 3 Risk of bias assessment. Image 3

## References

[1] Shi B., Ying T., Chadban S.J. (2023). Survival after kidney transplantation compared with ongoing dialysis for people over 70 years of age: a matched-pair analysis. Am J Transplant.

[2] Tonelli M., Wiebe N., Knoll G. (2011). Systematic review: kidney transplantation compared with Dialysis in clinically relevant outcomes. Am J Transplant.

[3] Barboza A.B., Dhanani N.H., Browning K., Wood R.P., Hall D.R. (2023). Trends in donation after circulatory determination of death donor utilization: lessons from Houston. Transplantation Reports.

[4] NHS Blood and Transplant (2024). How long is the wait for a kidney?. https://www.nhsbt.nhs.uk/organ-transplantation/kidney/receiving-a-kidney/how-long-is-the-wait-for-a-kidney/.

[5] Assembly Parliamentary (2003). Trafficking in organs in Europe. Published June.

[6] Wang J.H., Hart A. (2021). Global perspective on kidney transplantation: United States. Kidney360.

[7] Bellini M.I., Courtney A.E., McCaughan J.A. (2020). Living donor kidney transplantation improves graft and recipient survival in patients with multiple kidney transplants. J Clin Med.

[8] Fu R., Sekercioglu N., Berta W., Coyte P.C. (2020). Cost-effectiveness of deceased-donor renal transplant versus Dialysis to treat end-stage renal disease: a systematic review. Transplant Direct.

[9] De La Mata Nicole L., Khou Victor, Hedley James A., Kelly Patrick J., Morton Rachael L., Wyburn Kate (2023). Journey to kidney transplantation: patient dynamics, suspensions, transplantation and deaths in the Australian kidney transplant waitlist. Nephrol Dial Transplant.

[10] Chan G.C.K., Chow K.M. (2020). Should we use kidneys from donors with acute kidney injury for renal transplantation?. Nephrology.

[11] Koyawala N., Parikh C.R. (2020). A review of donor acute kidney injury and Posttransplant outcomes. Transplantation.

[12] Rao S.L. (2017).

[13] Kayler L.K. (2009). Outcomes and utilization of kidneys from deceased donors with acute kidney injury. Am J Transplant Off J Am Soc Transplant Am Soc Transplant Surg.

[14] Ugarte R.K. (2005). Excellent outcomes after transplantation of deceased donor kidneys with high terminal creatinine and mild pathologic lesions. Transplantation.

[15] Benck U.S. (2015). Excellent graft and patient survival after renal transplantation from donors after brain death with acute kidney injury: a case-control study. Int Urol Nephrol.

[16] Molina M.A. (2015). Results of kidney transplantation from deceased donors with acute kidney injury. Transplant Proc.

[17] Algeizawi S. (2011).

[18] Bauer J.G. (2018). Success of kidney transplantations from deceased donors with acute kidney injury. Ann Transplant.

[19] Domagala P.G. (2019). Successful transplantation of kidneys from deceased donors with terminal acute kidney injury. Ren Fail.

[20] Farney A.C. (2013). Evolving experience using kidneys from deceased donors with terminal acute kidney injury. J Am Coll Surg.

[21] Heilman R.L. (2015 Aug). (2015). Transplanting kidneys from deceased donors with severe acute kidney injury. Am J Transplant.

[22] Heilman R.L. (2012).

[23] Aull M., S S. (2015). 2015 American transplant congress.

[24] Mancuso A., N Z. (2022). http://ovidsp.ovid.com/ovidweb.cgi?T=JS&PAGE=reference&D=emed23&NEWS=N&AN=637181176.

[25] Stratta R.F. (2021). http://ovidsp.ovid.com/ovidweb.cgi?T=JS&PAGE=reference&D=emed22&NEWS=N&AN=636331025.

[26] Zuckerman J.M. (2009). Single center experience transplanting kidneys from deceased donors with terminal acute renal failure. Surgery.

[27] Kwon J.A., Park H., Park S.J. (2019). Factors of acute kidney injury donors affecting outcomes of kidney transplantation from deceased donors. Transplant Proc.

[28] Pei J., Cho Y., See Y.P. (2021). Impact of deceased donor with acute kidney injury on subsequent kidney transplant outcomes–an ANZDATA registry analysis. PLoS One.

[29] Boffa C., van de Leemkolk F., Curnow E. (2017). Transplantation of kidneys from donors with acute kidney injury: friend or foe?. Am J Transplant.

[30] Schütte-Nütgen K., Finke M., Ehlert S. (2019). Expanding the donor pool in kidney transplantation: should organs with acute kidney injury be accepted? - a retrospective study. PLoS One.

[31] Lenain R. (2022). Association between deceased donor acute kidney injury assessed using baseline serum creatinine Back-estimation and graft survival: results from the French national CRISTAL registry. Am J Kidney Dis.

[32] Nita G.E., Gopal J.P., Khambalia H.A., Moinuddin Z., van Dellen D. (2023). Kidney transplantation from donors with acute kidney injury: are the concerns justified? A systematic review and Meta-analysis. Transpl Int.

[33] Larpparisuth N., Nivatvongs S. (2023). Impact of acute kidney injury and renal recovery status in deceased donor to kidney transplant outcome: results from the Thai national transplant registry. Sci Rep.

[34] Rodrigo E.M. (2010). Using RIFLE criteria to evaluate acute kidney injury in brain-deceased kidney donors. Nephrology, dialysis, transplantation : official publication of the European Dialysis and Transplant Association - European Renal Association.

[35] Yuan X.P. (2014). Kidney transplantation from cardiac death donors with terminal acute renal failure. Transplant Proc.

[36] Jung C.W. (2013). Clinical outcomes in kidney transplantation patients from deceased donors with acute kidney injury. Transplant Proc.

[37] Jiang Y.S. (2019). Single kidney transplantation from donors with acute kidney injury: a single-center experience. Pediatr Transplant.

[38] Kim K.L. (2021). Safety and effectiveness of kidney transplantation using a donation after brain death donor with acute kidney injury: a retrospective cohort study. Sci Rep.

[39] Yu M.Y. (2018). Trend, not severity, of acute kidney injury affects graft outcome in deceased donor kidney transplantation. Clin Transpl.

[40] Fabian Echterdiek D.K. (2022). Outcome of kidney transplantations from ≥65-year-old deceased donors with acute kidney injury. Clin Transpl.

[41] Park W.C. (2019). Impact of acute kidney injury in expanded criteria deceased donors on post-transplant clinical outcomes: multicenter cohort study. BMC Nephrol.

[42] Ali T., W D. (2015). Outcomes of kidneys utilized from deceased donors with severe acute kidney injury. QJM.

[43] Cima L.N.L. (2019). Histopathology and long-term outcome of kidneys transplanted from donors with severe acute kidney injury. Prog Transplant.

[44] Hall I.E.P. (2015). Associations of deceased donor kidney injury with kidney discard and function after transplantation. Am J Transplant Off J Am Soc Transplant Am Soc Transplant Surg.

[45] Heilman R.L., Smith M.L. (2019). Long-term outcomes following kidney transplantation from donors with acute kidney injury. Transplantation.

[46] Lee M.H. (2014). Clinical outcome of kidney transplantation from deceased donors with acute kidney injury by acute kidney injury network criteria. J Crit Care.

[47] Schütte-Nütgen K.F. (2019). Expanding the donor pool in kidney transplantation: should organs with acute kidney injury be accepted?—a retrospective study. PLoS One.

[48] Gwon J.G. (2018). Clinical outcomes in kidney transplantation from deceased donors with acute kidney injury based on acute kidney injury network criteria. Transplant Proc.

[49] Liu C., Hall I.E. (2020). Association of Deceased Donor Acute Kidney Injury with Recipient Graft Survival. JAMA Netw Open.

[50] Ha J., Jung C.W. (2021). Impact of acute kidney injury on graft outcomes of deceased donor kidney transplantation: a nationwide registry-based matched cohort study in Korea. PLoS One.

[51] Srinivas T.R. (2010). Outcomes of renal transplantation. In: comprehensive clinical nephrology. Elsevier.

[52] Li M.T., Ramakrishnan A., Yu M. (2023). Effects of delayed graft function on transplant outcomes: a Meta-analysis. Transplant Direct.

[53] Gill J., Dong J., Rose C., Gill J.S. (2016). The risk of allograft failure and the survival benefit of kidney transplantation are complicated by delayed graft function. Kidney Int.

[54] Coemans M., Süsal C., Döhler B. (2018). Analyses of the short- and long-term graft survival after kidney transplantation in Europe between 1986 and 2015. Kidney Int.

[55] Gopalakrishnan G., Gourabathini S. (2007). Marginal kidney donor. Indian Journal of Urology.

[56] Miglinas M., Supranaviciene L. (2013). Delayed graft function: risk factors and the effects of early function and graft survival. Transplant Proc.

[57] Le Dinh H., Weekers L., Bonvoisin C. (2012). Delayed graft function does not harm the future of donation-after-cardiac death in kidney transplantation. Transplant Proc.

[58] Phillips B.L., Ibrahim M. (2021). Effect of delayed graft function on longer-term outcomes after kidney transplantation from donation after circulatory death donors in the United Kingdom: a national cohort study. Am J Transplant.

[59] Lim W.H., Johnson D.W. (2019). Association between duration of delayed graft function, acute rejection, and allograft outcome after deceased donor kidney transplantation. Transplantation.

[60] Lasserre J., Arnold S. (2012). Predicting the outcome of renal transplantation. J Am Med Inform Assoc.

[61] Helal I., Abderrahim E., Ben Hamida F. (2009). The first year renal function as a predictor of long-term graft survival after kidney transplantation. Transplant Proc.

[62] Naesens M., Budde K., Hilbrands L. (2022). Surrogate endpoints for late kidney transplantation failure. Transpl Int.

[63] Alshaikh E.A., Astor B.C., Muth B. (2023). Delayed graft function among kidney transplant recipients is associated with an increased risk of urinary tract infection and bk viremia. Transplant Direct.

[64] Guimarães-Souza N.K., Dalboni M.A., Câmara N.C. (2010). Infectious complications after deceased kidney donor transplantation. Transplant Proc.

[65] Salvadori M., Tsalouchos A. (2022). Innovative immunosuppression in kidney transplantation: a challenge for unmet needs. World J Transplant.

[66] Choudhary D., Sharma A., Singh S. (2022). Application of ex vivo normothermic machine perfusion in deceased donors with acute kidney injury with successful renal transplantation: a preliminary experience. Transplant Direct.

[67] Meier-Kriesche H.U., Schold J.D. (2005). The impact of pretransplant dialysis on outcomes in renal transplantation. Semin Dial.

[68] Nakagawa K., Tang J.F. (2011). Physiologic response of human brain death and the use of vasopressin for successful organ transplantation. J Clin Anesth.

[69] Parikh C.R., Puthumana J., Shlipak M.G. (2017). Relationship of kidney injury biomarkers with long-term cardiovascular outcomes after cardiac surgery. J Am Soc Nephrol.

[70] Belcher J.M., Sanyal A.J., Peixoto A.J., TRIBE-AKI Consortium (2014). Kidney biomarkers and differential diagnosis of patients with cirrhosis and acute kidney injury. Hepatology.

[71] Dupont M., Shrestha K., Singh D. (2012). Lack of significant renal tubular injury despite acute kidney injury in acute decompensated heart failure. Eur J Heart Fail.

[72] Meier-Kriesche H.U., Schold J.D. (2005). The impact of pretransplant dialysis on outcomes in renal transplantation. Semin Dial.

