# Supplementary material for: Impact of deceased donor acute kidney injury (AKI) on renal transplant outcomes
Source: Surg Open Sci. 2025 Nov 24;29:7–21. doi: 10.1016/j.sopen.2025.11.001 (PMC12771102; doi:10.1016/j.sopen.2025.11.001)
Supplement: Supplementary file 1 — Appendix 1 Acute Kidney injury classifications. [file mmc1.pdf]

Supplemental digital Content

Appendix 1: Acute Kidney injury classifications.

RIFLE criteria are the oldest, first introduced in 2004, used to diagnose AKI, and to categorise its severity into five stages: Risk, Injury, Failure, Loss, and End-stage kidney disease (RIFLE)[34].

1. The Risk stage signifies a 1.5-fold increase in serum creatinine or a decrease in glomerular filtration rate (GFR) by 25% or less.
2. Injury denotes a twofold increase in serum creatinine or a decrease in GFR by 50%.
3. Failure stage marks a threefold increase in serum creatinine or a GFR decrease of 75% or more or the initiation of renal replacement therapy.
4. Loss stage implies persistent AKI, which involves complete loss of kidney function for more than four weeks.
5. End-stage kidney disease stage is characterized by end-stage renal failure, lasting more than three months.

The AKIN (Acute Kidney Injury Network) criteria were introduced in 2007 to provide a standardised framework for diagnosing and classifying AKI [42]. These criteria categorise AKI into three stages based on changes in serum creatinine levels and urine output:

1. Stage 1 involves an increase in serum creatinine by 1.5 times the baseline within 7 days or a urine output of less than 0.5 mL/kg/h for 6 to 12 hours.
2. Stage 2 signifies a serum creatinine increase of 2.0 to 2.9 times the baseline or a urine output less than 0.5 mL/kg/h for over

   12 hours.
3. Stage 3 denotes a serum creatinine rise of three times the baseline, a serum creatinine of 4.0 mg/dL or more, or initiation of renal replacement therapy, among other criteria.

The Kidney Disease Improving Global Outcomes (KDIGO) criteria for AKI were introduced in 2012 as an evolution of previous classifications.

These criteria categorise AKI into three stages based on changes in serum creatinine levels and urine output:

1. Stage 1 is defined as an increase in serum creatinine by 1.5 to 1.9 times the baseline within 7 days or a urine output less than

0.5 mL/kg/h for 6 to 12 hours.

2. Stage 2 denotes a serum creatinine increase of 2.0 to 2.9 times the baseline or a urine output less than 0.5 mL/kg/h for over

12 hours.

3. Stage 3 signifies a serum creatinine rise of three times the baseline or more, a serum creatinine of 4.0 mg/dL or more, or initiation of renal replacement therapy, among other criteria.
