# Supplementary material for: Impact of deceased donor acute kidney injury (AKI) on renal transplant outcomes
Source: Surg Open Sci. 2025 Nov 24;29:7–21. doi: 10.1016/j.sopen.2025.11.001 (PMC12771102; doi:10.1016/j.sopen.2025.11.001)
Supplement: Supplementary file 2 — Appendix 2 Embase & Ovid MEDLINE(R) Search strategy [file mmc2.pdf]

Appendix 2

Embase & Ovid MEDLINE(R) Search strategy

1. (acute kidney injury or AKI or (kidn* and injur*)).mp. [mp=ti, ab, hw, tn, ot, dm, mf, dv, kf, fx, dq, nm, ox, px, rx, ui, sy]

2. exp acute kidney failure/

3. 1 or 2

4. ((DCD or donation after circulatory death or DBD or donation after brain death) and donor and (kidn* or renal)).mp. [mp=ti, ab, hw, tn, ot, dm, mf, dv, kf, fx, dq, nm, ox, px, rx, ui, sy]

5. exp kidney donor/

6. 4 or 5

7. 3 and 6

8. (surv* or dgf or delayed graft function or creat*).mp. or *GFR/ or glomerular filtration rate.mp. or PNF.mp. or primary non-function.mp. or protein*.mp. or acute rejection.mp. or infect*.mp. [mp=ti, ab, hw, tn, ot, dm, mf, dv, kf, fx, dq, nm, ox, px, rx, ui, sy]

9. exp graft survival/

10. exp kidney graft rejection/ or exp delayed graft function/

11. exp glomerulus filtration rate/

12. albumin to creatinine ratio/ or creatinine/ or protein creatinine ratio/ or creatinine clearance/

13. exp acute graft rejection/

14. exp surgical infection/ and infection/

15. 8 or 9 or 10 or 11 or 12 or 13 or 14

16. 7 and 15

17. 16

18. limit 17 to english language
