# Supplementary material for: Impact of deceased donor acute kidney injury (AKI) on renal transplant outcomes
Source: Surg Open Sci. 2025 Nov 24;29:7–21. doi: 10.1016/j.sopen.2025.11.001 (PMC12771102; doi:10.1016/j.sopen.2025.11.001)
Supplement: Supplementary file 3 — Appendix 3 Risk of bias assessment. [file mmc3.pdf]

Appendix 3: Risk of bias assessment.

Reference number	Country of study	Name of the paper	1. Was the resarch question clearly stated?	2. Was the study population clearly specified and defined?	3. Was the participation of eligible persons at least 50%?	4. Were all the subjects selected or recruited from the same or similar populations (including the same time period)? Were inclusion and exclusion criteria for being in the study prespecified and applied uniformly to all participants?	5. Was a sample size justification, power description, or variance and effect estimates provided?	6. For the analyses in this paper, were the exposure(s) of interest measured prior to the outcome(s) being measured?	7. Was the timeframe sufficient so that one could reasonably expect to see an association between exposure and outcome if it existed?	8. For exposures that can vary in amount or level, did the study examine different levels of the exposure as related to the outcome (e.g., categories of exposure, or exposure measured as continuous variable)?	9. Were the exposure measures (independent variables) clearly defined, valid, reliable, and implemented consistently across all study participants?	10. Was the exposure(s) assessed more than once over time?	11. Were the outcome measures (dependent variables) clearly defined, valid, reliable, and implemented consistently across all study participants?	12. Were the outcome assessors blinded to the exposure status of participants?	13. Was loss to follow-up after baseline 20% or less?	14. Were key potential confounding variables measured and adjusted statistically for their impact on the relationship between exposure(s) and outcome(s)?	Quality Rating (Good, Fair or Poor)
Algeizawi, S. &. (2011).	USA	A single center experience utilizing kidneys from deceased donors with low terminal estimated creatinine clearances. [Abstract]	No	Yes	Yes	Yes	No	Yes	Yes	Yes	Yes	No	Yes	N/A study format	N/A - no specific information	No	Fair
Ali T., D. W. (2015).	Saudi Arabia	Outcomes of kidneys utilized from deceased donors with severe acute kidney injury	Yes	Yes	Not stated	Yes	No	Yes	Yes	Yes	Yes	No	Yes	N/A study format	N/A - no specific information	No	Fair
Bauer, J. G. (2018)	Germany	Success of Kidney Transplantations from Deceased Donors with Acute Kidney Injury	Yes	Yes	Yes	Yes	No	Yes	Yes	Yes	Yes	No	Yes	N/A study format	N/A - no specific information	Yes	Good
Benck, U. S. (2015)	Germany	Excellent graft and patient survival after renal transplantation from donors after brain death with acute kidney injury: a case–control study	Yes	Yes	Not stated	Yes	No	Yes	Yes	Yes	Yes	No	Yes	N/A study format	N/A - no specific information	Yes	Fair
Cima, L. N.-L. (2019).	Italy	Histopathology and LongTerm Outcome of Kidneys Transplanted From Donors With Severe Acute Kidney Injury	Yes	Yes	Not stated	Yes	No	Yes:	Yes	Yes	Yes	No	Yes	N/A study format	N/A - no specific information	No	Fair
Domagala, P. G. (2019).	Poland	Successful transplantation of kidneys from deceased donors with terminal acute kidney injury	Yes	Yes	Not stated	Yes	No	No	Yes	Yes	Yes	No	Yes	N/A study format	N/A - no specific information	Yes	Fair
Fabian Echterdiek, D. K. (2022).	Germany	Outcome of kidney transplantations from ≥65-year-old deceased donors with acute kidney injury	Yes	Yes	Not stated	Yes	No	Yes	Yes	Yes	Yes	No	Yes	N/A study format	Yes	Yes	Good

Farney, A. C. (2013)	US	Evolving Experience Using Kidneys from Deceased Donors with Terminal Acute Kidney Injury	No	Yes	Not stated	Yes	No	Yes	Yes	Yes	Yes	No	Yes	N/A study format	N/A - no specific information	No	Poor
Gwon, J. G. (2018)	South Korea	Clinical Outcomes in Kidney Transplantation from Deceased Donors with Acute Kidney Injury Based on Acute Kidney Injury Network Criteria	Yes	Yes	Not stated	Yes	No	Yes	Yes	Yes	Yes	No	Yes	N/A study format	N/A - no specific information	No	Fair
Hall, I. E.-P. (2015).	USA	Associations of Deceased Donor Kidney Injury With Kidney Discard and Function After Transplantation	Yes	Yes	Not stated	Yes	No	Yes	Yes	Yes	Yes	Yes	Yes	N/A study format	N/A - no specific information	Yes	Good
Heilman, R. L. (2015)	USA	Transplanting Kidneys from Deceased Donors With Severe Acute Kidney Injury	Yes	Yes	Not stated	Yes	No	Yes	Yes	Yes	Yes	No	Yes	Partial blinding of the reviewing pathologist	N/A - no specific information	Yes	Good
Heilman, R. L. (2019).	USA	Long-term Outcomes Following Kidney Transplantation From Donors With Acute Kidney Injury	Yes	Yes	Not stated	Yes	No	Yes	Yes	Yes	Yes	No	Yes	N/A study format	N/A - no specific information	Yes	Fair
Heilman, R. L., Smith, M., Chakkera, H., Khamash, H., Hamawi, K., Moss, A., . . . Reddy, K. S. (2012).	USA	Impact of using deceased donor kidneys with acute kidney injury (AKI) on 1year protocol biopsy findings. [Abstract]	Yes	Yes	Yes	Yes	No	Yes	Yes	Yes	yes	Yes	Yes	N/A study format	N/A - no specific information	yes	fair
Jiang, Y. S. (2019).	China	Single kidney transplantation from donors with acute kidney injury: A single-center experience	Yes	Yes	Not stated	Yes	No	Yes	Yes	Yes	Yes	No	Yes	N/A study format	N/A - no specific information	Yes	Good
Jung, C. W. (2013)	South Korea	Clinical Outcomes in Kidney Transplantation Patients From Deceased Donors With Acute Kidney Injury	Yes	Yes	Not stated	Yes	No	Yes	Yes	Yes	Yes	No	Yes	N/A study format	N/A - no specific information	Yes	Fair
Kayler, L. K. (2009).	USA	Outcomes and Utilization of Kidneys from Deceased Donors with Acute Kidney Injury	Yes	Yes	Not stated	Yes	No	Yes	Yes	Yes	Yes	No	Yes	N/A study format	N/A - no specific information	Yes	Good
Kim, K. L. (2021)	South Korea	Safety and effectiveness of kidney transplantation using a donation after brain death donor with acute kidney injury: a retrospective cohort study	Yes	Yes	Not stated	Yes	No	Yes	Yes	Yes	Yes	No	Yes	N/A study format	N/A - no specific information	Yes	Good
Kwon, J. A. (2019).	South Korea	Factors of Acute Kidney Injury Donors Affecting Outcomes of Kidney Transplantation From Deceased Donors	Yes	Yes	Not stated	Yes	No	Yes	Yes	Yes	Yes	No	Yes	N/A study format	N/A - no specific information	Yes	Fair

Lee, M. H. (2014).	South Korea	Clinical outcome of kidney transplantation from deceased donors with acute kidney injury by Acute Kidney Injury Network criteria	Yes	Yes	Yes	Yes	No	Yes	Yes	Yes	Yes	Yes	Yes	N/A study format	N/A - no specific information	Yes	Good
M. Aull, S. S. (2015)	USA	Utilization of kidneys from deceased donors with acute kidney injury. [Abstract]	No	yes	yes	yes	no	yes	yes	yes	yes	yes	yes	N/A study format	N/A - no specific information	yes	Fair
Mancuso A., Z. N. (2022).	USA	Effect of Acute Kidney Injury and Prolonged Cold Ischemia Time on Kidney Transplant Outcomes.[Abstract]	No	Yes	Yes	Yes	Yes	Yes	Yes	yes	yes	yes	yes	N/A study format	N/A - no specific information	No	Fair
Molina, M. A. (2015).	Spain	Results of Kidney Transplantation From Deceased Donors With Acute	Yes	Yes	Not stated	Yes	No	Yes	Yes	Yes	Yes	Yes	No	N/A study format	N/A - no specific information	No	Poor
Park, W. C. (2019).	South Korea	Kidney Injury	Yes	Yes	Not stated	Yes	No	Yes	Yes	Yes	Yes	No	Yes	N/A study format	N/A - no specific information	Yes	Good
Rao S., L. I. (2017).	USA	Can kidneys from deceased donors with AKI and circulatory death be transplanted? [Abstract]	Yes	Yes	Yes	Yes	N/a- abstract	yes	yes	yes	yes	yes	yes	N/A study format	yes	yes	Good
Rodrigo, E. M.-F. (2010).	Spain	Using RIFLE criteria to evaluate acute kidney injury in brain-deceased kidney donors. [Abstract]	Yes	Yes	Not stated	Yes	No	Yes	Yes	Yes	Yes	Yes	Yes	N/A study format	N/A - no specific information	Yes	Good
Schütte-Nütgen, K. F. (2019).	Germany	Expanding the donor pool in kidney transplantation: Should organs with acute kidney injury be accepted? — A retrospective study	Yes	Yes	Not stated	Yes	No	Yes	Yes	Yes	Yes	Yes	Yes	N/A study format	N/A - no specific information	Yes	Good
Stratta R., F. A.-D.G. (2021).	USA	Long term outcomes of kidney transplantation from deceased donors with terminal acute kidney injury. [Abstract]	Yes	Yes	Yes	Yes	No	Yes	Yes	Yes	Yes	Yes	Yes	N/A study format	not stated	not stated	Fair
Ugarte, R. K. (2005).	USA	Excellent Outcomes after Transplantation of Deceased Donor Kidneys with High Terminal Creatinine and Mild Pathologic Lesions	Yes	Yes	Not stated	Yes	No	Yes	Yes	Yes	Yes	No	Yes	N/A study format	Not stated	Yes	Fair
Yu, M. Y. (2018).	South Korea	Trend, not severity, of acute kidney injury affects graft outcome in deceased donor kidney transplantation	Yes	Yes	Not stated	Yes	No	Yes	Yes	Yes	Yes	Yes	Yes	N/A study format	Not stated	Yes	Fair
Yuan, X. P. (2014).	China	Kidney Transplantation From Cardiac Death Donors With Terminal Acute Renal Failure	Yes	Yes	Not stated	Yes	No	Yes	Yes	Yes	Yes	No	Yes	N/A study format	Not stated	Yes	Fair

Zuckerman, J. M. (2009).	USA	Single center experience transplanting kidneys from deceased donors with terminal acute renal failure	Yes	Yes	Not stated	Yes	No	Yes	Yes	No	Yes	Yes	Yes	N/A study format	Not stated	Yes	Good
